# Comprehensive analysis of m6A methylated modification of fibrotic atria in rats induced by chronic intermittent hypoxia

**DOI:** 10.3389/fcvm.2025.1670859

**Published:** 2025-10-15

**Authors:** Tao Geng, Shiyu Qi, Xuan Cao, Jiao Li, Xiaodong Xia, Tianshu Gu, Hualing Wang, Pengyu Sun, Siyu Guan, Wenfeng Shangguan, Weiding Wang, Hao Zhang, Zhiqiang Zhao, Lijun Wang, Xue Liang

**Affiliations:** ^1^ Department of Cardiology, Cangzhou Central Hospital Affiliated to Hebei Medical University, Cangzhou, Hebei, China; ^2^ Tianjin Key Laboratory of Ionic-Molecular Function of Cardiovascular Disease, Department of Cardiology, Tianjin Institute of Cardiology, The Second Hospital of Tianjin Medical University, Tianjin, China; ^3^ Department of Cardiology, Tianjin Union Medical Center, The First Affiliated Hospital of Nankai University, Tianjin, China; ^4^ Department of Emergency Medicine, Tianjin Medical University General Hospital, Tianjin, China; ^5^ Medical School of Tianjin University, Tianjin University, Tianjin, China

**Keywords:** MeRIP sequencing, RNA sequencing, chronic intermittent hypoxia, atrial fibrosis, m6A methylation

## Abstract

**Background:**

Atrial fibrosis serves as a key pathological basis for atrial fibrillation, significantly elevating the risk of cardiovascular events. However, its molecular mechanisms remain incompletely understood. N⁶-methyladenosine (m6A) modifications have been proven to involve in the pathological processes of cardiovascular diseases, yet its role in atrial fibrosis remains unclear. m6A plays an important role in disease pathogenesis via mRNA modification. This study aimed to define the role of m6A modifications in the fibrotic atria of rats with chronic intermittent hypoxia (CIH).

**Methods:**

A CIH model was established using rats living in an intermittent hypoxia simulation chamber filled with oxygen and nitrogen. Myocardial function and atrial fibrosis were examined by echocardiography, electrophysiology, and histopathology. Methylated RNA immunoprecipitation sequencing (MeRIP-Seq) and mRNA sequencing (mRNA-Seq) were performed on atria from control and CIH rats to identify differential m6A methylated genes and transcripts and further analyze their coexistence. Functional enrichment of the conjoint genes was analyzed using Gene Ontology and Kyoto Encyclopedia of Genes and Genomes assays. m6A distribution of the conjoint gene ANGPTL4 (angiopoietin like 4) was also observed. ANGPTL4 and m6A-related gene expression levels were determined by quantitative real-time polymerase chain reaction.

**Results:**

CIH led to electrical conduction dysfunction and abnormal expression of fibrosis-associated proteins, indicating successful atrial fibrosis. Conjoint analysis identified 10 genes with upregulated m6A peaks and transcripts and 24 genes with downregulated m6A peaks and transcripts. These genes were functionally enriched in the calcium ion transport-related and fibrosis pathways (extracellular matrix receptor interaction). The m6A modification level of ANGPTL4 mRNA and the expression of four m6A regulatory enzymes were significantly different between control and CIH rats.

**Conclusion:**

Our results revealed that m6A modification plays a crucial role in atrial fibrosis and may provide new therapeutic strategies for this disease.

## 1 Background

Atrial fibrosis is recognized as a vital pathophysiological contributor and is characterized by the abnormal activation, proliferation, and differentiation of fibroblasts accompanied by excessive deposition and synthesis of myocardial extracellular matrix (ECM) proteins (1). Atrial fibrosis is a key biological event in cardiovascular disease, as it is a risk factor for atrial fibrillation (AF), a common arrhythmia that can lead to ischemic stroke and heart failure and increase disability and mortality (2). Pathologically, fibrosis is involved in the electrical and structural remodeling of AF and is closely related to abnormalities in cardiac structure and function. An increase in the degree of fibrosis leads to changes in atrial electrophysiological characteristics, thereby increasing the risk and recurrence rate of AF (3). However, the molecular mechanisms underlying atrial fibrillation remain unclear.

Recently, an increasing number of studies have focused on epigenetics due to its role in disease occurrence and the progression of diseases (4). Epigenetics primarily include DNA methylation, histone labeling, and RNA modifications, among which adenosine N(6) methylation (m6A) is the most common modification of eukaryotic messenger RNA (mRNA). m6A methylation is a dynamic and reversible process regulated by enzymes such as methyltransferases (writers), demethylases (erasers), and methylation reading proteins (readers) that are responsible for m6A modification, removal, and recognition of m6A modification, respectively (5). m6A methylation is involved in the translation, degradation, splicing, and export of mRNA and widely affects biological processes in mammalian organisms such as development, cell differentiation, immunity, metabolism, and tumorigenesis (6). Accumulating evidence suggests that m6A modifications are involved in the pathological development of cardiovascular diseases. For example, one review summarized the evidence for the involvement of m6A methylation in the occurrence and development of heart failure (7). Research using a mouse cardiac ischemia-reperfusion model revealed that the m6A methyltransferase METTL14 (methyltransferase 14, N6-adenosine-methyltransferase non-catalytic subunit) protects cardiomyocytes against ischemia-reperfusion injury (8). In the context of myocardial fibrosis, the m6A methyltransferase METTL3 (methyltransferase 3, N6-adenosine-methyltransferase complex catalytic subunit) was observed to control myocardial hypertrophy and fibrosis in cardiomyocytes (9–11). However, the involvement of m6A methylation in the pathology of atrial fibrosis remains largely unknown.

In this study, we performed methylated RNA immunoprecipitation sequencing (MeRIP-Seq) on the atrial tissues of control and chronic intermittent hypoxia (CIH) rats, an animal model that has been demonstrated to induce atrial fibrosis (12), and we identified differential m6A peaks (including 105 genes with upregulated hypermethylated m6A and 28 genes with downregulated hypermethylated m6A). Further conjoint analysis of differentially expressed m6A peak-annotated genes and transcripts suggested that m6A methylation was associated with calcium ion transport-related pathways and multiple fibrosis-related pathways.

## 2 Methods

### 2.1 CIH rat model

Eighty adult male SD rats (8 weeks old) were randomly divided into control and CIH groups (*n* = 40 per group). Rats in the CIH group lived in an intermittent hypoxia-simulated chamber for 8 h per day for 12 weeks. The chamber was alternately filled with oxygen (1.5 min) and nitrogen (3.5 min) every 5 min during each cycle. The oxygen concentration in the intermittent hypoxia simulation chamber was maintained between 7% and 21%. With the exception of the time spent in the simulation chamber, the living and feeding conditions of rats in both groups were the same. Twelve weeks after modeling, cardiac electrophysiological examination was performed, and the atria were isolated for fibrosis protein expression detection and high-throughput sequencing. The animals were fed and operated according to the Guiding Principles in the Care and Use of Animals (China) and in a manner approved by the Laboratory Animal Ethics Committee of Tianjin Medical University (No. TMUaMEC2016012).

### 2.2 Intracardiac electrophysiological examination

Rats were anesthetized by intraperitoneal injection of 1.5% tribromoethanol at a dosage of 300 mg/kg, and their limbs and heads were fixed on the operating table. A standard 2-lead body surface electrocardiogram (ECG) needle electrode was connected to record surface ECGs form the rats. The neck skin of the rats was cut, and the right jugular vein was exposed by blunt separation of the surrounding tissue. A multipolar electrophysiological catheter was slowly inserted into the right ventricle by puncturing the jugular vein. The multipolar electrophysiological catheter included eight round 0.5 mm electrodes spaced 0.5 mm apart (STG3008-FA, ADInstruments, New South Wales, Australia). The position of the multipole catheter was adjusted using ECG monitoring to ensure the successful pacing of the stimulation. Additionally, the stimulation voltage was selected to be twice the pacing threshold to ensure atrial pacing. For the AF induction rate evaluation scheme, continuous high-frequency stimulation was used as a burst stimulus, and the pacing cycle was set to start at 40 ms and then decrease to 20 ms (repeated five times). This was the burst stimulus. After burst stimulation, a 1 min rest was required to ensure that the rat atrium returned to the resting state. Successfully induced AF was defined as a burst of irregular non-sinus rhythms for >1 s with irregular RR intervals and irregular f waves on surface ECGs. If AF was induced three times among the five inductions, the rats were recorded as positive for AF.

### 2.3 Electrophysiological mapping of epicardium

At the end of the cardiac electrophysiology experiment, the trachea was intubated using the tracheotomy method and connected to a small animal ventilator. After the respiratory rate of the rats stabilized, the chest cavity was opened, and the heart was fully exposed by separating the pericardium and surrounding tissue. A 6 × 6 probe with 36 microelectrodes was gently attached to the surface of the left atrium, and the electrical activity of the microelectrodes was recorded using MappingLab EMapRecord 5 software. The conduction velocity (CV) and absolute inhomogeneity index (inhomogeneity index) were measured using MappingLab EMapScope 4 software. CV refers to the ratio of the distance between the earliest and last activation sites to the conduction time and is typically used to express the epicardial conduction velocity. Absolute conduction heterogeneity and heterogeneity index reflect the degree of electrical conduction uniformity in the atrial epicardium.

### 2.4 Immunohistochemistry

Rats were euthanized by intraperitoneal injection of 3% pentobarbital sodium solution at a dose of 100 mg/kg body weight. Atria were embedded in paraffin and then cut into 5 μm sections. After deparaffinization, the sections were immersed in citrate buffer and heated for 15 min, and this was followed by the addition of 3% hydrogen peroxide. Sections were immersed in blocking solution for 10 min and then reacted sequentially with primary antibody (overnight, 4°C) and secondary antibody (10 min, room temperature). The primary and secondary antibodies used are listed in Table 1. The sections were stained using 3,3′-diaminobenzidine chromogenic solution and hematoxylin staining solution. The sectioned tissue was dehydrated, drip-added to a neutral resin, and covered with a coverslip. Target protein staining was observed under a microscope (OLYMPUS, Japan).

**Table 1:** Primary and secondary antibodies used in immunohistochemical assay and western blot.

Primary antibodies	dilution (IHC/WB)	solvent (IHC/WB)
Rabbit Anti-Collagen I antibody (ab270993)	1: 500/1:1,000	PBS/TBST
Rabbit Anti-Collagen III antibody (ab7778)	1:200/1:5,000	PBS/TBST
Rabbit Anti-CTGF (ab227180)	1:100/1:1,000	PBS/TBST
Rabbit anti-TGF-β1 (ab170874)	1:150/1:1,000	PBS/TBST
Mouse anti-MMP2 (ab86607)	1:200/1:1,000	PBS/TBST
Rabbit anti-MMP9 (ab76003)	1:1,000/1:5,000	PBS/TBST
Mouse anti-α-SMA (ab7817)	1:200/1:1,000	PBS/TBST
Rabbit anti-POSTN (ab92460)	1:200/1:1,000	PBS/TBST
Mouse anti-β-actin (ab8226)	-/1:5,000	-/TBST
Secondary antibodies	Dilution (IHC/WB)	Solvent (IHC/WB)
Goat anti-rabbit IgG-HRP (ab7090)	1:5,000	PBS/TBST
Goat anti-mouse IgG-HRP (ab97040)	1:5,000	PBS/TBST

CTGF, connective tissue growth factor; HRP, horseradish peroxidase; IHC, immunohistochemistry; MMP, matrix metalloproteinase; PBS, phosphate buffer saline; POSTN, periostin; SMA, smooth muscle actin; TBST, Tris buffered saline tween; TGF, transforming growth factor.

### 2.5 Western blot

Total protein samples were prepared from the atria using RIPA lysis buffer (CWBIO, China) mixed with 1% PMSF. After the cell lysates were centrifuged, the supernatant containing protein was collected and boiled at 95°C for 10 min. The protein samples were subjected to 10%–15% SDS–PAGE and transferred to a polyvinylidene fluoride membrane (Millipore, USA). Subsequently, nonspecific proteins in the membrane were blocked with 5% nonfat milk, and the target proteins were immunoreacted with primary antibody overnight at 4°C. The membrane was then immersed in a horseradish peroxidase-conjugated secondary antibody for 2 h and subsequently immersed in ECL chemiluminescence liquid (Dingguo Changsheng Biotechnology, Beijing, China). Table 1 lists the primary and secondary antibodies used in this study. The visualized immunoblots of the target proteins were photographed, and their relative expression was analyzed using β-actin as an internal reference.

### 2.6 MeRIP sequencing

Atria from 30 control rats and 30 CIH rats were subjected to RNA extraction (the atria of every 10 rats were used as test samples), and the extracted RNA was used for MeRIP and RNA sequencing. Total RNA samples were prepared using the Invitrogen TRizol™ Reagent (Thermo Fisher, USA). The RNA quality was examined using an Agilent 2,100 (Agilent Technologies, USA). A total of 10 μg of RNA was mixed with RNA Fragmentation Reagents (Invitrogen, USA) and reacted for 10 min at 70°C to break RNA into fragments of approximately 100 nt. RNA fragments were incubated with a bead-m6A antibody for 4 h for immunoprecipitation using a magna-methylated RNA immunoprecipitation (MeRIP) m6A kit (Merck Millipore, USA). The immunoprecipitated RNA fragments were eluted from the beads, purified with phenol:chloroform:isoamyl alcohol (125:24:1) (Sigma-Aldrich, USA), and subjected to library preparation and high-throughput sequencing by RiboBio (Guangzhou, China) using an Illumina PE150 model on a NovaSeq 6,000 sequencer (Illumina, USA).

The peak calling and differential m6A peak identification were conducted using Package “macs2/exomePeak” (Version 3.8) software. The m6A-modified regions (termed peaks in m6A-IP samples) were analyzed with the corresponding m6A-input samples as the controls. The differential m6A peak was identified as the peak with *P* < 0.05 and |logFC|>1 and annotated to the RefSeq database (hg19/mm10/m6) using STAR software (version 2.5.3a, Cold Spring Harbor Laboratory, USA) with default parameters. Peak annotation and the m6A binding motif were analyzed using HOMER (version 4.9) (13) and DREME (14). The m6A peaks were visualized using an integrative genomics viewer (IGV) (15) according to the BW file of the sequencing samples. Prediction score distribution along the query sequence was calculated using a sequence-based RNA adenosine methylation site predictor (SRAMP) algorithm (16).

### 2.7 RNA sequencing

A total of 2 μg of RNAs were used for library preparation for digital mRNA sequencing (mRNA-Seq) using the Ribo-off rRNA Depletion Kit (Human/Mouse/Rat) (Illumina) and the KC-Digital™ Stranded mRNA Library Prep Kit for Illumina® (Wuhan Seqhealth Co., Ltd, China). Library products (200–500 bp) were quantified and subjected to high-throughput sequencing using an Illumina PE150 model on a Novaseq 6,000 sequencer (Illumina). The differentially expressed transcript was defined as the cutoff of *P* < 0.05 between the CIH group and the control group according to *t*-test. Heatmaps and volcano plots were constructed for genes with differential m6A peaks and transcripts using ggplot2 software (R foundation, USA). The genes with differential m6A peaks were annotated and received conjoint analysis with the differentially expressed transcript of mRNA-seq to discern the shared genes. The potential functions of the shared genes were explored by Gene Ontology (GO) and Kyoto Encyclopedia of Genes and Genomes (KEGG) functional enrichment analyses using KOBAS3.0 (Peking University, Beijing, China).

### 2.8 Quantitative real-time polymerase chain reaction (qRT-PCR)

RNA samples were prepared from the atria of control and CIH rats using TRIzol reagent (Thermo Fisher Scientific, USA). RNA quantity was evaluated using a spectrophotometer. The RNA was reverse transcribed using an RT Reagent Kit with gDNA Eraser (TaKaRa, Japan), and the generated cDNA was subjected to real-time PCR using SYBR Green PCR Master Mix (Thermo Fisher Scientific). The primers used are listed in Table 2. The PCR procedure included 40 cycles of denaturation at 95°C for 15 s and annealing and extension at 60°C for 30 s. Relative expression of each mRNA was calculated using the housekeeping gene glyceraldehyde-3-phosphate dehydrogenase as the internal control as per the 2^–ΔΔCt^ method.

**Table 2:** Primers used in real-time PCR.

Primer	Species	Sequence (5′-3’)	Tm (℃)
GAPDH	Rat	F: AAATGGTGAAGGTCGGTGTGAAC	58.0
		R: CAACAATCTCCACTTTGCCACTG	
METTL3	Rat	F: CCTCAGATGTTGACCTGGAGATAG	58.0
		R: GACTGTTCCTTGGCTGTTGTG	
METTL14	Rat	F: GATCGCAGCACCTCGGTCATT	58.0
		R: CCCACTTTCGCAAACATACTCTCC	
WTAP	Rat	F: TTCAAACGATGTGACTGGCTTA	58.0
		R: TCTCCTGTTCCTTGGTTGCTA	
FTO	Rat	F: TGTGGAAGAAGATGGAGAGTGTGA	58.0
		R: GGATCAGGACGGCAGACAGAA	
ALKBH3	Rat	F: TCCGCAACCAAGACTTACAG	58.0
		R: ACAGGAAGCCAGTGAGGAT	
ALKBH5	Rat	F: GGGTTCTTATGTTCTTGGCTTTCC	58.0
		R: ATCTCTACTGGCTACTCTGGTGT	
ANGPTL4	Rat	F: GGGACCTTAACTGTGCCAAGA	58.0
		R: CCGTTGCCGTGGAATAGAGT	

GAPDH, glyceraldehyde-3-phosphate dehydrogenase; METTL3, methyltransferase 3, N6-adenosine-methyltransferase complex catalytic subunit; METTL14, methyltransferase 14, N6-adenosine-methyltransferase non-catalytic subunit; WTAP, WT1 associated protein; FTO, FTO alpha-ketoglutarate dependent dioxygenase; ALKBH3, alkB homolog 3, alpha-ketoglutarate dependent dioxygenase; ALKBH5, alkB homolog 5, alpha-ketoglutarate dependent dioxygenase; ANGPTL4, angiopoietin like 4.

### 2.9 Statistical analysis

Data are expressed as the means ± standard error. Statistical evaluations were performed using SPSS 22.0 software, and graphs were drawn using GraphPad Prism version 9.0 (GraphPad Software). The differences between the control and CIH rats were tested using the Student's *t*-test, and the AF induction rate was compared using the chi-square test in both groups. A *p*-value of less than 0.05 represents a notable difference between groups.

## 3 Results

### 3.1 CIH induces changes in cardiac function and atrial fibrosis in rats

**Figure 1:**
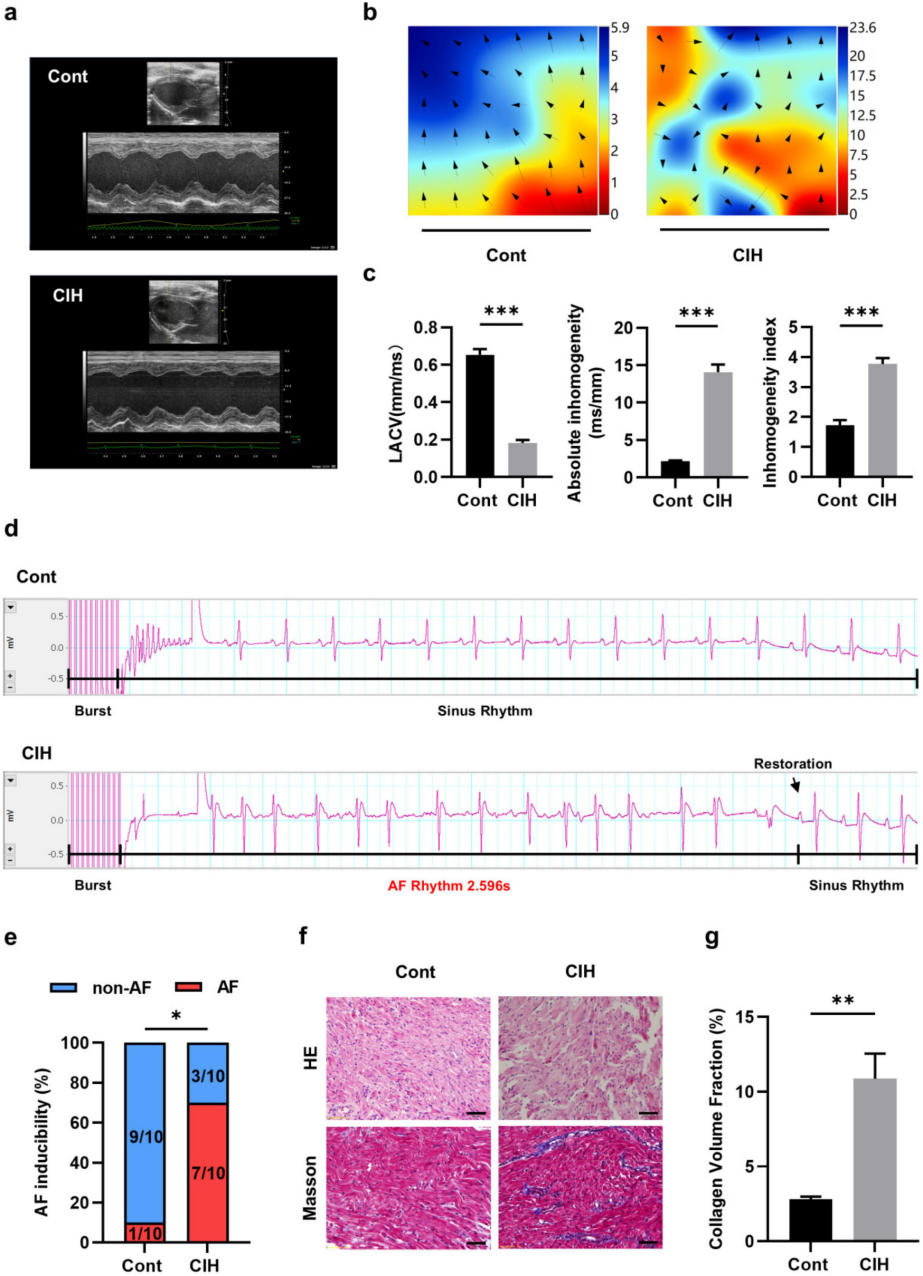
Characteristics of atrial fibrosis in a CIH rat model. **(a)** Typical echocardiographic images of two groups of rats. **(b)** Typical diagram of electrical conduction in the epicardial membrane of the left atrium. **(c)** Statistical results for left atrial conduction velocity, absolute heterogeneity, and heterogeneity index between the two groups (*N* = 5). ***P < 0.001. **(d)** Representative ECG of sinus rhythm and AF episodes after burst stimulation. **(e)** Statistical results for AF induction rate (*N* = 10). *P < 0.05. **(f)** Representative images stained with HE and Masson (400×). Scale = 50 μm. **(g)** Statistical results of atrial fibrosis collagen volume fraction in the two groups (*N* = 3). **P < 0.01. All data are expressed as mean ± standard error (SEM). The data between the two groups were compared using an unpaired *t*-test, and the AF induction rates were compared using the chi-square test.

**Figure 2:**
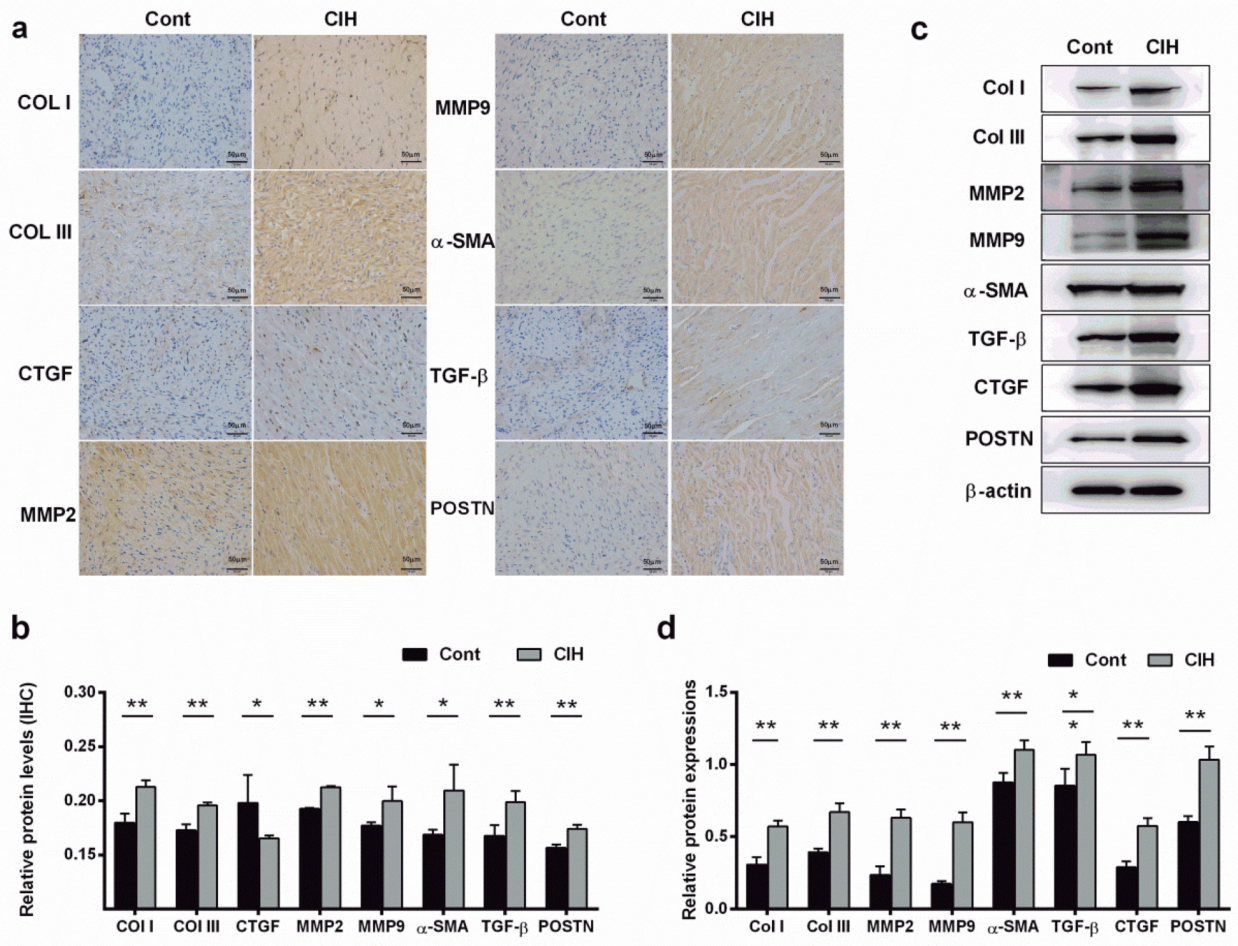
Fibrosis-related proteins are expressed in the fibrotic atrial tissue of CIH rats. **(a,b)**. Expression levels of fibrosis-related proteins were detected by immunohistochemistry **(a)** and quantified **(b)** (400×). **(c,d)** Expression levels of fibrosis-related proteins were detected by western blot **(c)** and quantified **(d)**. *P < 0.05, **P < 0.01. *N* = 3; All data are expressed as mean ± standard error (SEM). The data between the two groups were compared using an unpaired *t*-test.

Echocardiography results suggested that ventricular wall motion was significantly reduced in the CIH group (Figure 1a). Echocardiography and caudal artery blood pressure data demonstrated that left atrial diameter (LAD), left ventricular end-diastolic diameter (LVEDD), and left ventricular end-systolic diameter (LVESD) increased and interventricular septal thickness during diastole (IVS-D), interventricular septal thickness during systole (IVS-S), left ventricular posterior wall thickness during diastole (LVPW-D), and left ventricular posterior wall thickness during systole (LVPW-S) decreased in CIH rats (Table 3). The heart weight/tibia length (HW/TL) ratio in the control group was comparable to that in the CIH group was (*P* = 0.179), confirming that CIH did not induce significant cardiac hypertrophy in our model. The electrical conduction of the left atrial outer membrane in the control group spread in an orderly manner to the surrounding tissues, whereas that in the CIH group was disordered, and the conduction between the left atrial outer membrane and the surrounding tissues was not uniform (Figure 1b). In the statistical analysis, compared to that of control rats, the conduction velocity of the left atrial adventitia in the CIH rats was significantly decreased, whereas the absolute heterogeneity and heterogeneity index were significantly increased (Figure 1c), suggesting that CIH could lead to a decline in the electrical conduction function of the atrial adventitia in rats. The results of AF induction indicated that CIH rats exhibited a higher susceptibility to AF than did control rats (Figures 1d,e). Histopathological analysis demonstrated that the CIH group possessed a disordered atrial tissue structure and a higher degree of fibrosis (Figures 1f,g). Both IHC and western blot results indicated the protein expression levels of fibrosis-associated genes, including matrix metalloproteinase 2 (MMP2), MMP9, collagen type I (Col-I), Col-III, connective tissue growth factor (CTGF), α-smooth muscle actin (α-SMA), transforming growth factor-β (TGF-β), and periostin (POSTN), were significantly upregulated in atrial tissues of CIH rats (Figures 2a–d), further confirming that CIH induces atrial fibrosis.

**Table 3:** Rat echocardiography and caudal artery blood pressure data.

Variables	Cont (*n* = 15)	CIH (*n* = 15)	*P*
LAD (mm)	4.62 ± 0.40	5.00 ± 0.40*	0.005
IVS-D (mm)	2.04 ± 0.23	1.80 ± 0.13*	<0.001
IVS-S (mm)	3.13 ± 0.27	2.86 ± 0.27*	0.001
LVEDD (mm)	7.47 ± 0.59	7.84 ± 0.48*	0.032
LVESD (mm)	4.26 ± 0.66	4.92 ± 0.41*	0.001
LVPW-D (mm)	2.18 ± 0.26	2.01 ± 0.26*	0.044
LVPW-S (mm)	3.29 ± 0.35	2.98 ± 0.28*	0.004
LV_vol_-D (ul)	297.50 ± 50.11	333.08 ± 43.35*	0.021
LV_vol_-S (ul)	84.43 ± 23.39	114.76 ± 22.05*	<0.001
LVEF (%)	72.03 ± 5.92	65.61 ± 3.96*	0.001
FS (%)	42.89 ± 5.75	37.51 ± 3.13*	0.001
BP-S (mmHg)	111.10 ± 1.89	132.20 ± 2.61*	<0.001
BP-D (mmHg)	81.87 ± 3.50	105.90 ± 4.60*	0.002
mPAP (mmHg)	69.90 ± 2.23	66.63 ± 2.92*	0.002
HW/TL (mg/mm)	35.15 ± 0.47	34.94 ± 0.37	0.179

* *P* < 0.05 compared with Cont (*n* = 15).

BP-D, diastolic blood pressure; BP-S, systolic blood pressure; CIH, chronic intermittent hypoxia; Cont, control; FS, left ventricular fractional shortening; HW/TL, heart weight/tibia length; IVS-D, interventricular septal thickness during diastole; IVS-S, interventricular septal thickness during systole; LAD, left atrial diameter; LVEDD, left ventricular end-diastolic diameter; LVEF, left ventricular ejection fraction; LVESD, left ventricular end-systolic diameter; LVPW-D, left ventricular posterior wall thickness during diastole; LVPW-S, left ventricular posterior wall thickness during systole; LVvol-D, left ventricular volume during diastole; LVvol-S, left ventricular volume during systole; mPAP, mean pulmonary artery pressure.

### 3.2 Transcriptome-wide MeRIP-Seq reveals the overall characteristics of the m6A peak distributions in control and CIH rats

**Figure 3:**
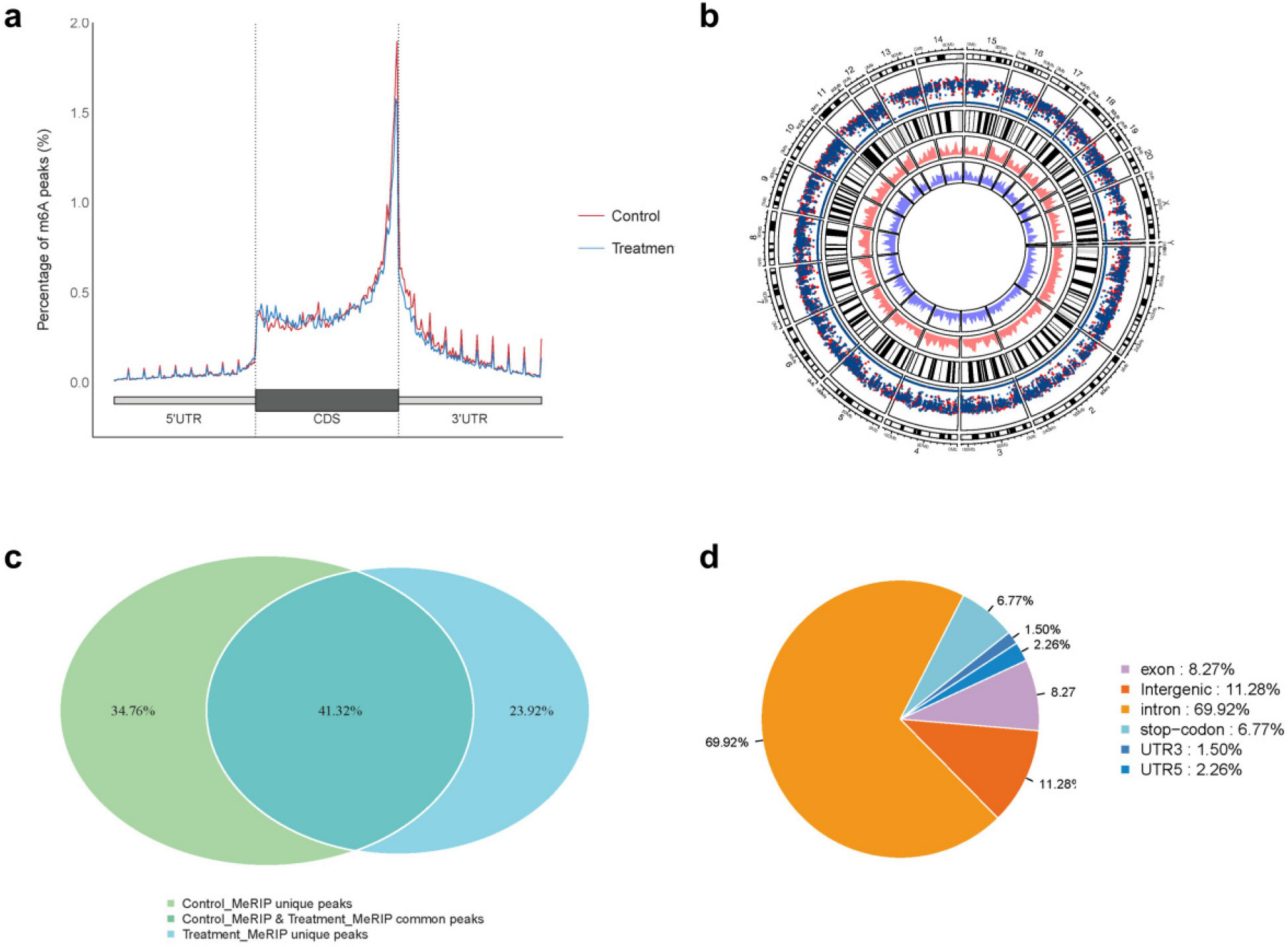
Differential m6A peak analysis between control and CIH rats. **(a)** m6A peak distributions in the 5′-UTR, CDS, and 3′UTR regions of mRNA. **(b)** Circos diagram displays the m6A profile over chromosomes. **(c)** Venn diagram indicates peaks shared by control and CIH rats. **(d)** Annotated distribution of differential m6A peaks in different gene contexts in CIH rats.

The sequencing data were aligned with the reference genome, and the number and length of m6A peaks were confirmed using TopHat software (v2.0.13) (Table 4). The overall length of the m6A peak varied from 1,173,899 bp to 3, 329, 809 bp in the control and CIH samples. m6A peaks were observably related to two distinct coordinates, including the start of the coding sequence (CDS) and the beginning of the 3′ untranslated region (3′UTR) (Figure 3a). The distribution of m6A peaks in the CIH rats and control rats exhibited the same trend, rising from the 5′ untranslated region (5′UTR) to the beginning of the 3′UTR and descending from the beginning of the 3′UTR to the end of the transcriptome. However, the density of m6A of CIH rats in the 3′UTR region was lower than that of the controls (Figure 3a). The m6A peaks were distributed across the chromosomes (Figure 3b). The control and CIH samples exhibited only 41.32% common peaks (Figure 3c). To methodically assess enrichment, we assigned each m6A peak to one of five non-overlapping transcript segments. As a result, 77.18% of m6A peaks were located within the coding region (introns and exons) (Figure 3d). The m6A motifs concentrated in rats of the control and CIH groups are listed in Table 5.

**Table 4:** The number and length of peaks across the controls and CIH samples.

Sample	PeakNum	AverageLength	MedianLength	TotalLength
Cont1_MeRIP	4,606	488.77	384	2,251,270
Cont2_MeRIP	3,098	405.85	305	1,257,322
Cont3_MeRIP	6,433	482.75	377	3,105,556
CIH1_MeRIP	6,779	491.19	382	3,329,809
CIH2_MeRIP	5,135	402.66	299	2,067,635
CIH3_MeRIP	3,177	369.50	376	1,173,899

**Table 5:** Motifs across m6A peaks in control and CIH rats.

Sample	Motif				
Cont1_MeRIP	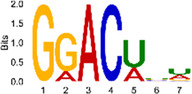	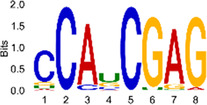	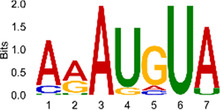	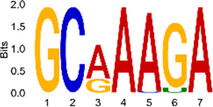	
Cont2_MeRIP	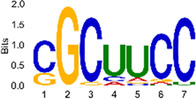	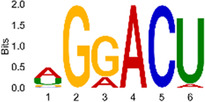	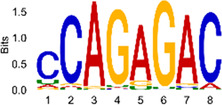	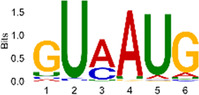	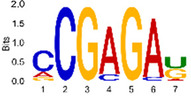
Cont3_MeRIP	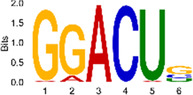	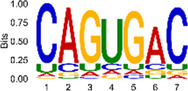	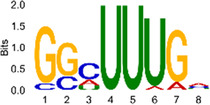	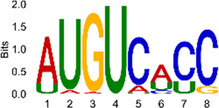	
CIH1_MeRIP	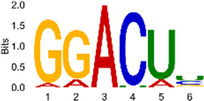	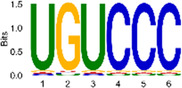	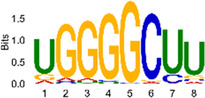	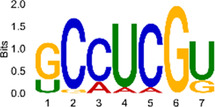	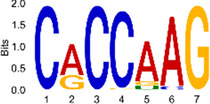
CIH2_MeRIP	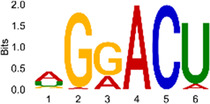	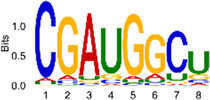	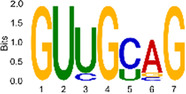	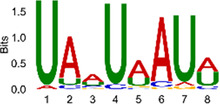	
CIH3_MeRIP	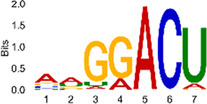	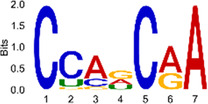	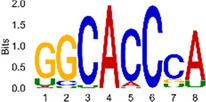	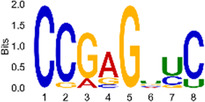	

### 3.3 Differential m6A peaks and transcripts were identified in fibrotic atria

**Figure 4:**
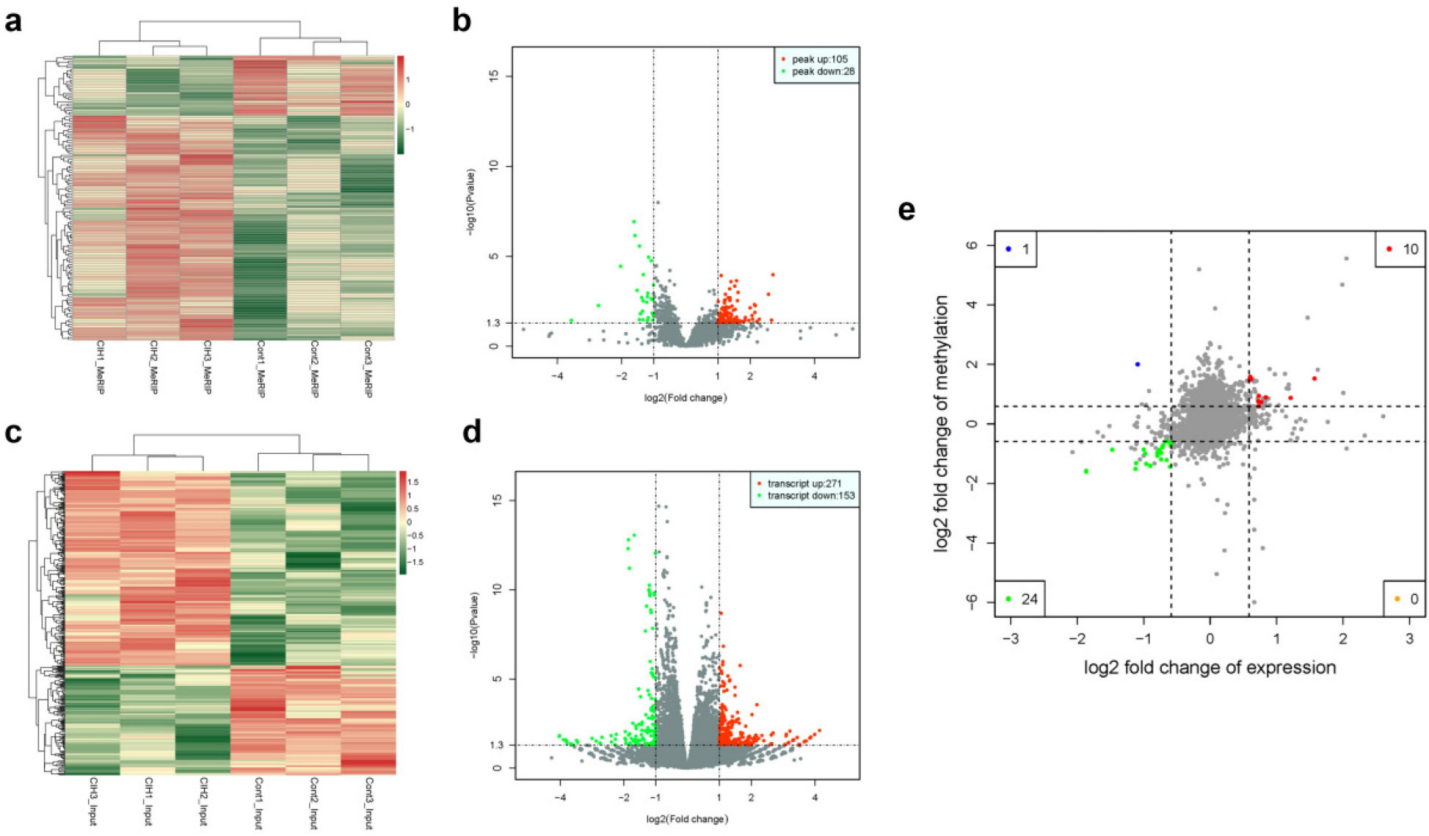
Differential m6A peaks and transcripts. **(a)** Heat map of differential m6A peaks. **(b)** Volcano plot of differential m6A peaks. **(c)** Heat map of differentially expressed transcripts. **(d)** Volcano plot of differentially expressed transcripts. **(e)** Conjoint analysis of genes with significant differential m6A peaks and differentially expressed transcripts. Blue and green spots represent significant upregulation or downregulation of m6A. Red and orange spots represent significant upregulation or downregulation of differentially expressed transcripts.

The heat map displayed differential m6A methylation peaks in CIH and control atrial tissues (Figure 4a) that included 105 significantly upregulated hyper-methylated m6A peaks and 28 significantly downregulated hypermethylated m6A peaks (fold change >1.3, *P* < 0.05) (Figure 4b). Table 6 presents the top 20 differential m6A peaks, including the upregulation of LOC100911417, Lyn, Sox5, Cdh20, Ophn1, Zfp654, Sncaip, RGD1304728, Cdh13 and Atrnl1 and the downregulation of Myh911, Myh9, Fgg, Foxe1, Fnip2, RGD1559575, Prokr2, Cpxm2, and Lrrn4. A heat map of differentially expressed transcripts between the control and CIH groups (Figure 4c) included 271 upregulated transcripts and 153 downregulated transcripts (fold change >1.3, *P* < 0.05) (Figure 4d). Conjoint analysis revealed the co-expression of genes with differential m6A modifications and differentially expressed transcripts, encompassing genes with simultaneous upregulation (10 genes, including Vash2, Angptl4, Slc25a34, Tubb3, Stmn3, Adgra1, LOC102550048, Uchl1, Adgra1, and Lnc215) (Table 7) or downregulation (24 genes, Table 8) and genes with opposite trends (Figure 4e).

**Table 6:** The top 20 differently methylated m6A peaks.

Gene	Gene ID	log_2_ (FC)	*p*-value	Chromsome	Start	End	Peak_length	Region
LOC100911417	100,911,417	5.55	2.96698E-32	Chr2	208,409,630	208,409,955	325	Intron
Lyn	81,515	2.71	0.000106108	chr5	16,552,092	16,552,319	227	Intron
Sox5	140,587	2.67	0.035706082	Chr4	178,465,843	178,466,068	225	Intron
Cdh20	363,948	2.57	0.001283526	chr13	24,679,373	24,679,641	268	Intron
Ophn1	312,108	2.29	0.031507343	chrX	68,411,946	68,412,172	226	Intron
Zfp654	288,354	2.21	0.041795912	chr11	1,860,053	1,860,351	298	Intron
Sncaip	307,309	2.16	0.005538060	chr18	47,784,314	47,784,521	207	Intron
RGD1304728	360,560	2.15	0.013126615	chr10	58,743,087	58,743,344	257	Intron
Cdh13	192,248	2.14	0.004824475	chr19	51,755,448	51,755,881	433	Intron
Atrnl1	307,992	2.08	0.031315912	chr1	278,758,829	278,759,088	259	Intron
Myh9l1	25,745	−5.99	0.028731061	chr7	118,771,251	118,771,452	201	Intron
Sphkap	316,561	−2.17	0.005460955	chr9	89,329,233	89,329,480	247	Intergenic
Myh9	69,709	−2.03	0.000035899	chr7	119,001,887	119,002,109	222	Exon
Fgg	24,367	−1.61	1.18247E-07	chr2	181,993,531	181,993,729	198	Stop-codon
Foxe1	192,274	−1.51	0.000780637	chr5	61,954,825	61,955,407	582	UTR5
Fnip2	310,538	−1.44	0.032957645	chr2	178,235,032	178,235,233	201	Intron
RGD1559575	287,231	−1.44	2.67012E-06	chr10	34,241,724	34,242,067	343	Exon
Prokr2	192,649	−1.41	0.015628763	chr3	125,009,082	125,009,317	235	Stop-codon
Cpxm2	293,566	−1.37	0.001974051	chr1	204,049,691	204,049,942	251	Stop-codon
Lrrn4	311,443	−1.34	0.011311799	chr3	125,522,949	125,523,654	705	UTR3

**Table 7:** Transcript and m6A methylation levels of upregulated genes in conjoint analysis of MeRIP-seq and RNA-seq.

Gene	m6A				Transcriptome	
	PeakID	log_2_FC	*p-value*	Region	log_2_FC	*p-value*
Vash2	diff_1479	1.58	0.0002282	Stop-codon	0.60	0.0012495
Angptl4	diff_813	1.52	0.0004499	Stop-codon	1.57	0.0007552
Slc25a34	diff_258	0.87	0.0007739	Exon	1.21	0.0098501
Tubb3	diff_2333	0.73	0.0008939	Intron	0.77	0.0105064
Stmn3	diff_2666	0.89	0.0012626	Exon	0.84	0.0005739
Adgra1	diff_16	0.94	0.0054963	Stop-codon	0.73	0.0001413
LOC102550048	diff_2271	1.48	0.0098805	Intergenic	0.59	0.0224413
Uchl1	diff_2765	0.75	0.0104990	Exon	0.73	0.0011816
Adgra1	diff_3233	0.59	0.0209435	Exon	0.73	0.0001413
Lnc215	diff_1740	1.49	0.0348945	Intron	0.62	0.0000654

**Table 8:** Transcript and m6A methylation levels of downregulated genes in conjoint analysis of MeRIP-seq and RNA-seq.

Gene	m6A				Transcriptome	
	PeakID	log_2_FC	*p-value*	Region	log_2_FC	*p-value*
LOC103689965	diff_1003	−0.86	1.02357E-08	Exon	−1.00	0.0004826
Fgg	diff_2713	−1.61	1.18247E-07	Stop-codon	−1.86	4.87849E-13
Fgg	diff_3621	−1.58	7.06631E-07	Intron	−1.86	4.87849E-13
RGD1559575	diff_159	−1.44	2.67012E-06	Exon	−0.60	0.0001878
Bicdl1	diff_124	−1.07	0.0000176	Exon	−0.79	5.45330E-07
Bicdl1	diff_1850	−0.94	0.0000359	Intron	−0.76	1.01410E-06
Nat8f3	diff_3209	−1.32	0.0001035	Stop-codon	−.1.11	2.66972E-06
Adgre1	diff_2634	−0.88	0.0002351	Intron	−0.76	1.20141E-11
Chst4	diff_105	−1.00	0.0003884	Exon	−0.97	8.31112E-09
Foxe1	diff_173	−1.51	0.0007806	UTR5	−1.12	0.0080070
RT1-A2	diff_2086	−0.64	0.0008495	Intron	−0.60	0.0000216
Bicdl1	diff_3477	−1.00	0.0012325	Exon	−0.79	5.45330E-07
Rspo1	diff_232	−0.72	0.0014418	UTR5	−0.71	0.0003025
RT1-A2	diff_2085	−0.73	0.0017619	Intron	−0.60	0.0000216
Chrdl1	diff_2955	−1.19	0.0017635	Intron	−0.73	5.16063E-06
Bicdl1	diff_723	−0.97	0.0018116	Exon	−0.79	5.45330E-07
Crb2	diff_3380	−1.21	0.0035803	Exon	−0.65	0.0020231
Anxa1	diff_1647	−0.59	0.0035881	Intron	−0.67	2.23240E-15
Lrrn4	diff_268	−1.34	0.0113118	UTR3	−0.96	0.0027670
Prokr2	diff_647	−1.41	0.0156288	Stop-codon	−0.90	0.0066046
Chrdl1	diff_2782	−0.97	0.0216582	Intron	−0.73	5.16063E-06
C6	diff_2496	−0.60	0.0283728	Intron	−0.65	1.80917E-08
Cd2	diff_1896	−0.79	0.0354323	Exon	−0.72	0.0458585
Gal3st2	diff_726	−0.87	0.0467916	Exon	−1.47	0.0000998

### 3.4 Functional enrichment analysis

**Figure 5:**
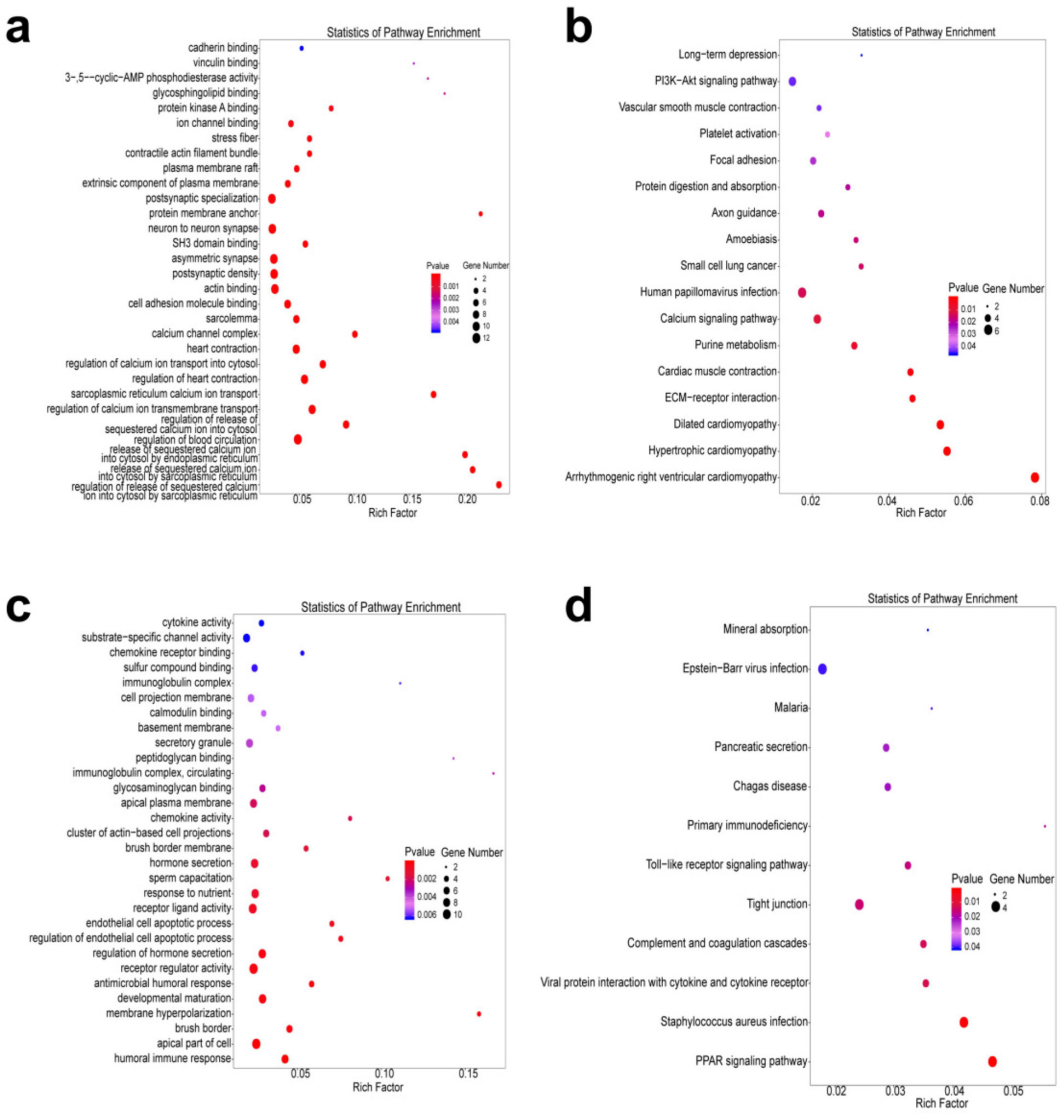
Functional enrichment analysis. **(a,b)** GO bubble chart **(a)** and KEGG bubble chart **(b)** of genes with increased m6A peaks. **(c,d)** GO bubble chart **(c)** and KEGG bubble chart **(d)** of differentially expressed transcripts. The size of the dots indicates the number of genes with differential m6A peaks or transcripts. The color of the dots corresponds to different *P* values.

To define the potential role of genes with differential m6A peaks and transcripts in CIH rats, functional enrichment of GO and KEGG pathways was observed. GO exploration revealed that the genes with differential m6A peaks in CIH rats were significantly related to calcium ion transport-related pathways such as calcium channel complex, regulation of calcium ion transport into the cytosol, and release of sequestered calcium ions into the cytosol by endoplasmic reticulum and sarcoplasmic reticulum (Figure 5a). KEGG pathway exploration revealed that these genes were significantly related to protein digestion and absorption, phosphatidylinositol—3—kinase—Protein kinase B (PI3K-AKT) signaling pathway, and ECM receptor interaction (Figure 5b). As for differentially expressed transcripts, GO exploration indicated that these genes were significantly enriched in the receptor regulatory response (Figure 5c), and KEGG pathway annotation demonstrated that these genes were relevant to the toll-like receptor and peroxisome proliferator—activated receptor (PPAR) signaling pathways (Figure 5d).

### 3.5 The visual representation of m6A

**Figure 6:**
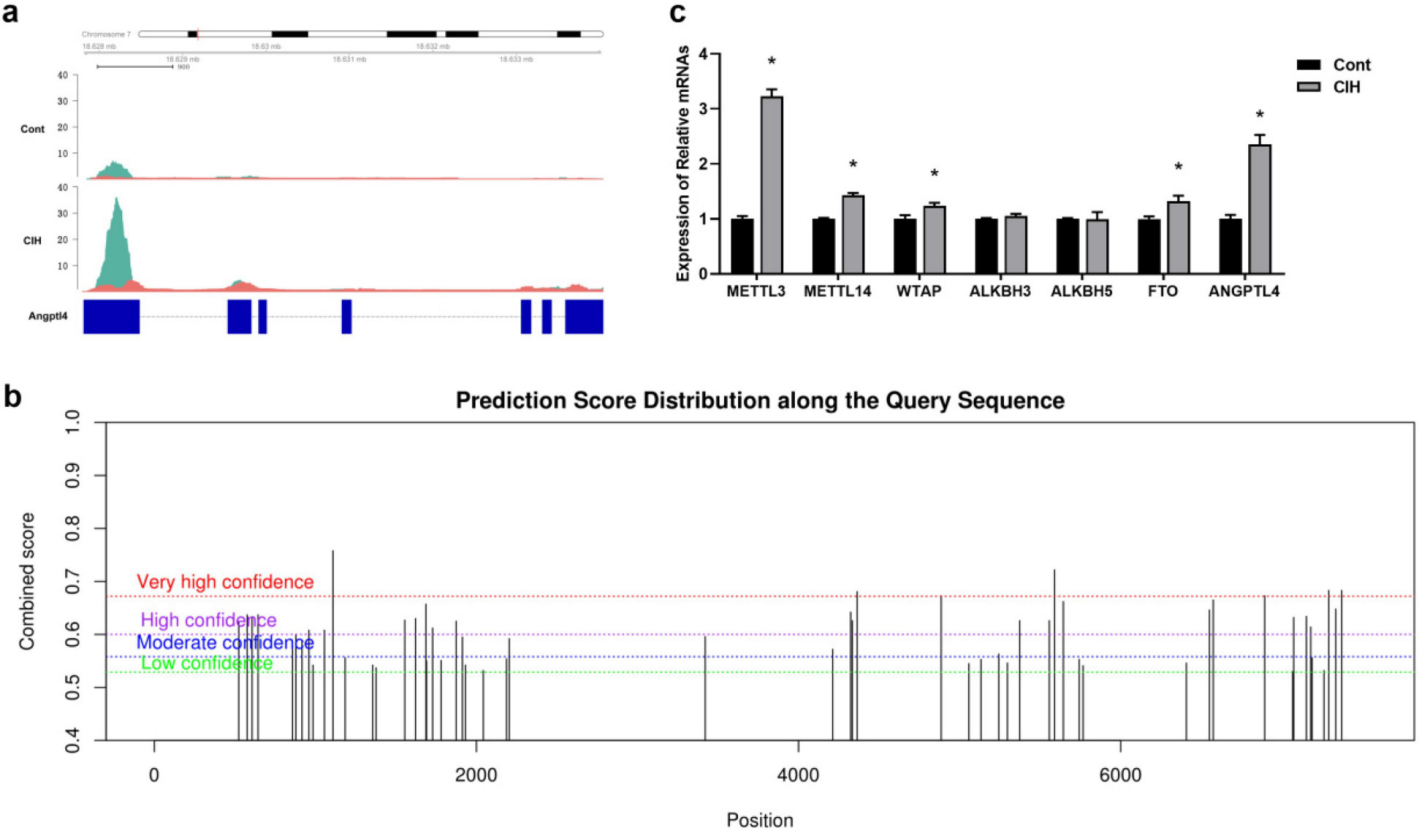
Validation of m6A modification in CIH rat atrial tissues. **(a)** Visualized tracks of m6A modifications of ANGPTL4 mRNA (located in Chromosome 7) in control and CIH rats using Integrative Genomics Viewer (IGV). **(b)** RNA adenosine methylation site predictor (SRAMP) algorithm analyzed the prediction score distributions along the query sequence. *Y* axis indicates the combined score at different levels of high (H), moderate (M), and low (L) probability. Vertical bars indicate the score for m6A sites. The *X*-axis indicates the Chromosome 7 genome in base pair resolution. **(c)** qRT-PCR was performed to determine the expression levels of six key regulatory enzymes that modulate dynamic m6A modification and ANGPTL4. *P < 0.05.

ANGPTL4 (angiopoietin like 4) that is located on chromosome 7 and appears to increase m6A peaks and transcripts in conjoint analysis was selected to verify m6A methylation distribution. As reported by IGV, the m6A modifications of ANGPTL4 mRNA differed between the control and CIH rats (Figure 6a). SRAMP identified 39 m6A sites in the ANGPTL4 mRNA sequence, with seven sites possessing the highest probability scores (Figure 6b). We further compared the expression of ANGPTL4 and six key regulatory enzymes that modulate dynamic m6A modifications in control and CIH rats. The results revealed that four m6A regulatory enzymes (METTL3, METTL14, WT1 associated protein (WTAP), and FTO (FTO alpha-ketoglutarate dependent dioxygenase)) and ANGPTL4 were upregulated in CIH rats, whereas no significant differences in the expression of two m6A regulatory enzymes, ALKBH3 (alkB homolog 3, alpha-ketoglutarate dependent dioxygenase) and ALKBH5, were observed (Figure 6c).

## 4 Discussion

Over the past few years, due to the ubiquitous m6A methylation of mRNA, its role in disease-related biological processes (including proliferation, metabolism, immunity, and apoptosis) has attracted wide attention. Accumulating evidence has revealed that m6A is associated with fibrosis. Specifically, the enzymes involved in the regulation of m6A improve or promote fibrosis progression by regulating the translation of target genes (particularly in the liver, kidney, lung, and myocardial tissues where fibrosis often occurs) (17–19). These findings highlight the important function of m6A in the biological progression of fibrosis and its potential as a therapeutic target. In the present study, based on MeRIP-Seq detection of atrial fibrosis tissues in CIH rats, we observed that high m6A methylation and low m6A methylation corresponded to high and low expression of transcripts, respectively, and these genes are related to the pathway of atrial fibrosis. We believe that revealing the relationship between m6A methylation and atrial fibrosis will help to clarify the pathological mechanism of atrial fibrosis and offer a potential basis for disease-modifying strategies.

m6A methylation occurs in coding sequences (CDS) and non-coding regions (3′UTR and 5′UTR) in eukaryotic mRNAs. When occurring in the 5′UTR and 3′UTR of mRNA, m6A is associated with the functional controls such as translation initiation, mRNA precursor cleavage, mRNA structural stability, and mRNA transport, while the m6A methylation modification in the CDS region is used to maintain mRNA stability (20). Our MeRIP-seq results yielded the largest proportion of m6A peaks in the CDS and the richest m6A peak in the stop codon region (a region close to the 3′UTR and CDS), and this is consistent with a previous report (21). As m6A is related to mRNA pre-splicing, nuclear export, and mRNA stability, m6A modification has been observed to be positively related to transcript abundance (22, 23). In this study, we identified 10 upregulated and 24 downregulated differentially expressed m6A peaks and transcripts using conjoint analysis. Overall, these results indicated the involvement of m6A in atrial fibrosis.

Functional analysis of genes with differential m6A peaks and transcripts between CIH and control rats indicated that the genes were functionally relevant to calcium ion transport-related pathways, the PI3K-AKT signaling pathway, ECM receptor interaction, receptor regulatory response, the toll-like receptor signaling pathway, and the PPAR signaling pathway, and this may constitute the potential mechanism of atrial fibrosis. The molecular mechanisms involved in atrial fibrosis are highly complex and involve multiple biological events such as Ca2+ homeostasis dysregulation, ECM dysregulation, and signal transduction (24). Abnormal Ca2+ processing and increased sarcoplasmic reticulum release rate are the main causes of arrhythmia in patients with AF, and the effect of Ca2+ changes on AF has also been reported (25, 26). Alterations in Ca2+ homeostasis have recently been proposed to play an important role in the pathogenesis of atrial fibrosis. When AF and fibrosis risk factors (such as hypertension and diabetes) change, Ca2+-handling dysfunction and calpain activity also change, and the maintenance of Ca2+ homeostasis helps to improve atrial remodeling and the incidence of AF (27, 28). In addition to Ca2+ homeostasis, ECM-receptor interaction, an important intracellular biological event that mediates the interaction between the ECM and cells and affects cell migration, adhesion, proliferation, differentiation, and other cellular activities, is also important during atrial fibrosis. Transmembrane proteins that mediate this process, primarily integrins, proteoglycans, and others, play a role in regulating atrial fibrosis (29, 30). Additionally, several signaling pathways have also been determined to be engaged in the process of atrial fibrosis (24). In the present study, we observed that genes with m6A modification were enriched in the PI3K-AKT signaling pathway and PPAR signaling pathway. The PI3K-AKT signaling pathway is a vital pathway involved in fibrosis stimulation and is active in the kidney, liver, and heart (31–33). Activation or inhibition of PI3K-AKT-mediated pathway components can result in varying effects on atrial fibrosis (34, 35). PPARs are a family of ligand-activated nuclear hormone receptors consisting of three homologous members, PPARα, PPARβ/δ, and PPARγ, that affect the expression of genes correlated with energy metabolism, cell development, and differentiation. In a study investigating atrial fibrosis, activation of PPARγ by pioglitazone can improve the atrial structural and electrophysiological remodeling caused by diabetes (36). PPAR*α* was inhibited in the AF mouse model induced by AngII. Activation of PPAR*α* by clofibric acid can reverse atrial fibrosis in mice (37). Taken together, these results suggest that m6A methylation is involved in the process of atrial fibrosis through a variety of mechanisms.

Interestingly, our results revealed that angiopoietin-like protein 4 (ANGPTL4), a multifunctional secreted protein, possesses multiple m6A modification sites and is significantly upregulated in rats subjected to CIH. ANGPTL4 participates in the modulation of lipid metabolism and angiogenesis in a variety of tissues and has been observed to play a role in cardiovascular diseases, with abnormal lipid metabolism as a risk factor (38). Accumulating evidence suggests an interaction between fibrosis regulatory factors and lipid metabolism (39). In recent years, ANGPTL4 has been observed to be significantly upregulated in the tissues of animal fibrosis models (including the kidneys and lungs) (40, 41). These results are consistent with our detection of ANGPTL4 expression in CIH tissues. In terms of mechanism, in a study based on a non-alcoholic steatohepatitis mouse model, ANGPTL4 deficiency could lead to the accumulation of free cholesterol, thereby promoting the progression of liver fibrosis (42). A study based on an AngII-induced atrial fibrosis cell model *in vitro* reported that ANGPTL4 may inhibit fibrosis via downregulating PPAR*γ*, AKT, and others (43). Therefore, the role of ANGPTL4 up-regulation in atrial fibrosis requires further exploration.

This study has several limitations that should be acknowledged. First, our research was conducted exclusively using a CIH-induced rat model of atrial fibrosis. While CIH is a well-validated model for this pathology (12), the findings may not fully generalize to other etiologies of atrial fibrosis, which warrants verification in multiple animal models in future studies. Second, we focused on transcriptomic-level changes in m6A modification and its correlation with gene expression in whole left atrial tissue, but lacked identification of the specific cell type involved in m6A methylation. Third, although we identified ANGPTL4 as a key m6A-modified gene upregulated in CIH induced atrial fibrosis, its cell-specific function was not explored. Fourth, the mechanism by which m6A modification regulates target gene expression was not explored in depth. Despite these limitations, our findings provide novel insights into the epitranscriptomic regulation of atrial fibrosis and lay a foundation for subsequent mechanistic and translational research.

## 5 Conclusions

In summary, the findings of the current study confirm that m6A methylation is essential for atrial fibrosis and is associated with the abnormal elevation of enzymes regulating m6A modification, including METTL3, METTL14, WTAP, and FTO, exerting a regulatory effect on fibrosis-related pathways, including calcium transport-related pathways, through target proteins. However, the specific targets and exact mechanisms of m6A modifications in atrial fibrosis require further investigation and verification.

## Data Availability

The datasets presented in this study can be found in online repositories. The name of the repository and accession number are as follows: NCBI Sequence Read Archive (SRA), https://www.ncbi.nlm.nih.gov/sra/, accession number PRJNA1175848.
