# Supplementary figures and images for: Comprehensive analysis of m6A methylated modification of fibrotic atria in rats induced by chronic intermittent hypoxia
Source: Front Cardiovasc Med. 2025 Oct 15;12:1670859. doi: 10.3389/fcvm.2025.1670859 (PMC12568672; doi:10.3389/fcvm.2025.1670859)

β-actin

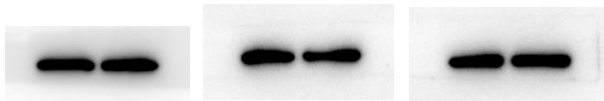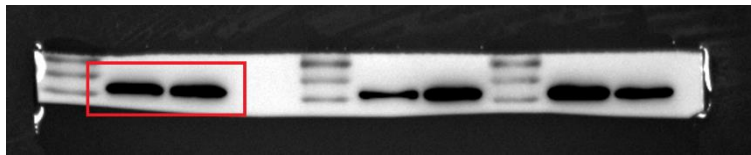

Col-I

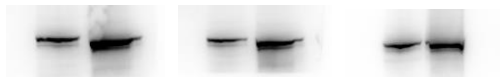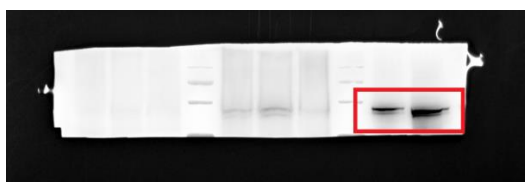

Col-III

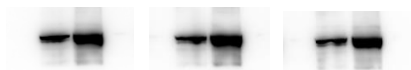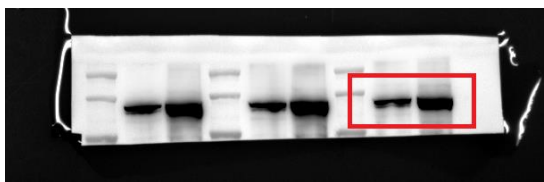

CTGF

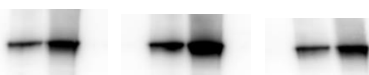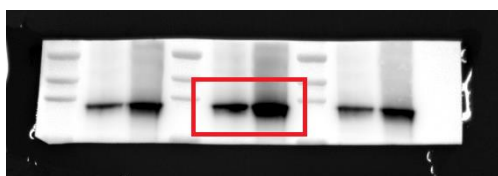

MMP2

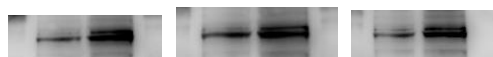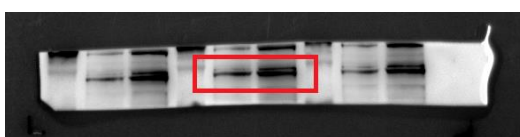

MMP9

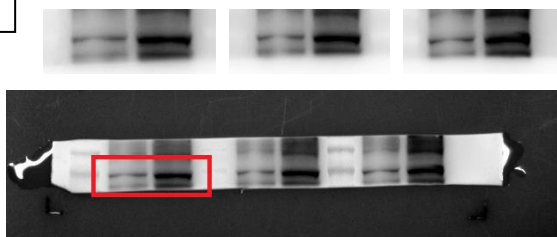

POSTN

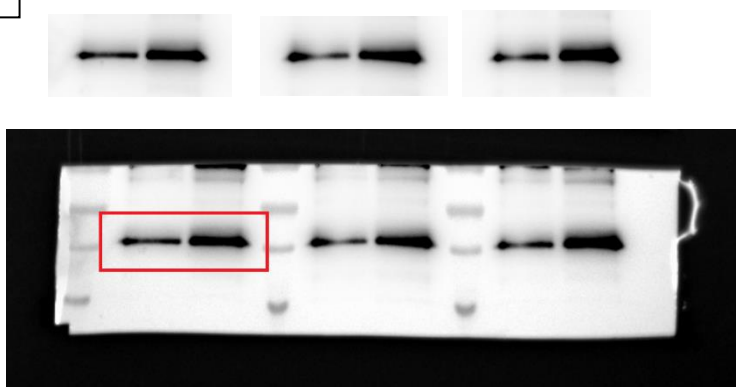

$\alpha$ -SMA

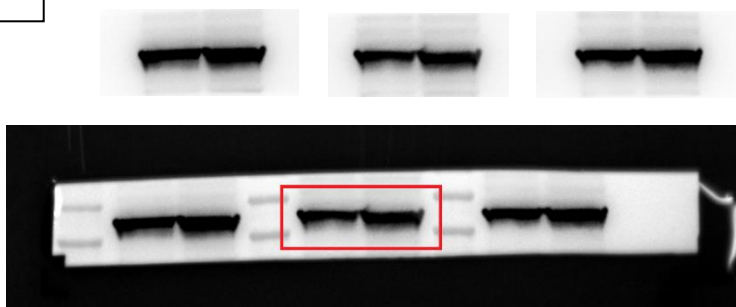

TGF- $\beta$

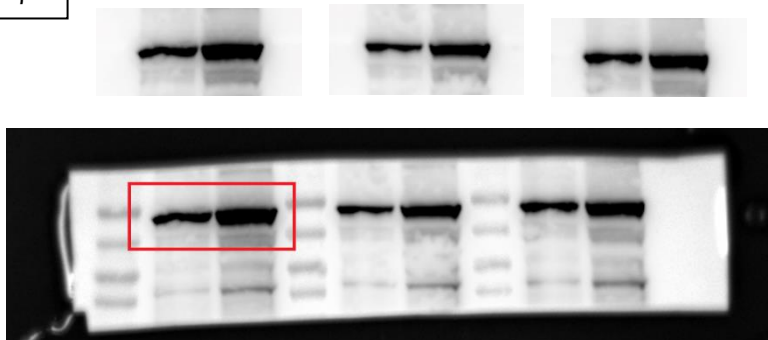

Supplement: Supplementary file 1 [file Datasheet1.pdf]
